# Geometry, Structure and Surface Quality of a Maraging Steel Milling Cutter Printed by Direct Metal Laser Melting

**DOI:** 10.3390/ma15030773

**Published:** 2022-01-20

**Authors:** Michal Skrzyniarz, Lukasz Nowakowski, Slawomir Blasiak

**Affiliations:** Department of Manufacturing Engineering and Metrology, Kielce University of Technology, 25314 Kielce, Poland; mskrzyniarz@tu.kielce.pl (M.S.); lukasn@tu.kielce.pl (L.N.)

**Keywords:** 3D printed cutting tool, four-flute end mill, additive manufacturing, prototyping

## Abstract

This article considers the use of additive manufacturing to produce cutting tools for various machining operations, especially turning, milling, and drilling. The right geometry and material of the tool as well as coatings applied on cutting edges are crucial as they improve the life and performance of the tool. The study described here focused on a four-flute end mill made of maraging steel 1.2709 using a Concept Laser M2 Cusing Direct Metal Laser Melting (DMLM) machine. Before the printed tool was first used, it was examined to determine its dimensional and geometric accuracy, surface roughness, and surface structure. The measurement data showed that the tool required machining, e.g., grinding, to improve its geometry because the total runout of the shank and the cutting edge radius were too high, amounting to 120 μm and 217 μm, respectively. The cutting edges were sharpened to obtain a fully functional cutting tool ready to perform milling operations. The study aimed to check the dimensional and geometric accuracy of the 3D printed milling cutter and determine the optimal machining allowance for its finishing.

## 1. Introduction

Additive manufacturing, also known as 3D printing, is an umbrella term for methods that involve layer-by-layer fabrication of objects. Although they have a relatively short history, with some of them being developed in the 1980s, there has been much progress in the field so far. Many different 3D printing technologies have emerged over the years, enabling the use of a wide range of materials. Today, 3D printing is increasingly replacing conventional manufacturing processes, especially machining, metal forming, injection molding, casting, welding, laser beam processing, and plasma processing. The most popular additive manufacturing methods include stereolithography (SLA) [1], fused deposition modeling (FDM), also called fused filament fabrication or filament freeform fabrication (FFF) [2], selective laser sintering (SLS) [3], laminated object manufacturing (LOM) [4], and 3D printing [5]. Analyzing the patent literature on the subject, one can find numerous ideas protected by copyright concerning, for example, the support material or the way it is removed [6].

Research on SLS includes a study by Kozior [7], which analyzes how the basic process parameters affect the model mass. He focuses on the density of the sintered material (polyamide PA 2200), stress relaxation under compression (determined in accordance with the ISO 3384 standard), and the surface texture parameters.

In [8,9], Bochnia et al. deal with the rheological properties of polymer specimens fabricated through SLS. The rheological data, i.e., stress relaxation and creep, predicted using the Maxwell–Wiechert and the Kelvin–Voigt models, respectively, were in agreement with the experimental results. In another study [10], they used a fractional order calculus to mathematically describe the material obtained through SLS.

Major advancements have been seen in prototyping mainly because there are more and more goods with short lifespans. The time to develop, commercialize, and produce them needs to be shortened as well. It is also essential to accelerate their inspection and testing, for instance, to determine their strength, wear resistance, and functionality so that manufacturers can survive or thrive in this hypercompetitive market. 3D printing technologies seem to meet all these requirements. There is no need for special tooling or additional equipment. 3D printers are being commonly used instead of conventional tools, for example, to create casting patterns or injection molds in small series production.

3D printing technologies use metal-, polymer-, and ceramic-based materials. With such a large range of materials, it is possible to create objects characterized by high strength and/or high dimensional and geometric accuracy [11], suitable for various industrial applications. 3D printing can also be used to rebuild, extend or join existing objects [12].

In [13], Asnafi et al. discuss methods to design and fabricate dies for sheet metal forming and plastic injection molding. In their experiments [14], they dealt with tools created using the Laser-based Powder Bed Fusion (LPBF) technology. They employed topology optimization to design their forming dies, which were additively manufactured with an LPBF 3D printer according to the DIN 1.2709 standard. The tools were certified to be used for forming of 2 mm thick steel metal sheets. The use of 3D printing reduced the lead time and improved the tool material efficiency; however, the cost of the tools and dies was much higher than the cost of their conventionally manufactured counterparts.

Studies on additively manufactured tools include those concerned with cutting tools. Velerga et al. [15] analyze the use of Reverse Engineering, which requires acquisition of data on the products made. 3D scanning, for instance, provides information about the geometry of an element, which can be used to generate its virtual model (VM) for further digital analysis. It is then possible to run functionality tests or create a full-size prototype. A product can thus be redesigned to be improved. In [16], Hückmann considers the use of 3D printing to fabricate tools with a geometry not available by means of conventional methods. It is emphasized that this new way of tool fabrication has many benefits. It is possible to increase the number of cutting edges and optimize their geometries using high performance cutting (HPC), which leads to higher cutting efficiency and shorter (up to 50%) cutting time. Additionally, the optimized shape and layout of special channels ensure precise supply of cutting fluid separately to each cutting edge. Lakner et al. [17] studied the supply of cutting fluid to the interface between the rake face and the chip. The purpose of their research was to increase the durability of cutting tools and apply higher cutting parameters to enhance the process efficiency. However, different mechanisms of tool wear were observed for different materials. Thus, the efficiency of supply of cutting fluid was largely dependent on the material cut.

Another problem examined for additively manufactured cutting tools is the influence of powder composition as well as particle size and geometry. In [18], Fortunato et al. analyze results concerning tools for machining WC-Co toothed wheels fabricated through selective laser melting (SLM). The same type of material was tested by Yang et al. [19]. They studied how the powder particle geometry and powder composition affected the SLM process. They fabricated and tested simple cylindrical specimens to determine the optimal process parameters and additive manufacturing strategies. The parameters were then used to produce a real cutting tool for machining toothed wheels.

Haeberle and Desai [20] analyze the suitability of AM (3D printing) for the design, prototyping, and development of rapid thermoform tooling. A Fused Deposition Modeling (FDM) 3D printer was used to fabricate thermoform tooling made of Polypropylene Styrofoam (PPSF). The analysis involved comparing an additively manufactured tool with a conventionally made tool using computerized numerical control (CNC) machines.

The study by Lakner et al. presented in [21] aimed to produce cutting tools with specially designed internal channels for supplying cutting fluid. The purpose of cutting fluid is to reduce the tool wear and chip formation. However, creating channels inside milling cutters to supply cutting fluid to the cutting edges is not an easy task. In traditionally produced milling tools, such channels are drilled in the material. The research focused on comparing the cutting fluid supply in the additively manufactured tool with that in a conventionally made tool.

Mikolajczyk et al. [22] propose an innovative design of a universal tool for machining complex planar and axially symmetric surfaces. Because of its geometric flexibility, the tool can be used to machine different shapes and surfaces in one setting. The general principle of operation of a tool with multiple cutting edges is that each cutting edge has a different role and the desired effect is achieved by combining these roles. The article discusses the design and applications of multiple-edge tools in turning and milling, depending on the edge combination.

In a series of articles, including [23], Zhu et al. describes a strategy for remanufacturing damaged gears, which involves the use of laser cladding. The analysis of a remanufactured element reveals that, although it may look like the original one, its mechanical properties have changed: the tensile strength is lower while the hardness and resistance to abrasive wear are higher. Laser cladding is also examined in [24], but focus is on the process simulation and the monitoring and optimization of the process parameters. The article reviews materials used in laser cladding, especially high-entropy alloys (HEAs), as well as amorphous and monocrystalline alloys. It also discusses the use of laser cladding to produce special-purpose coatings and repair machine parts.

There is not much research on the use of additive manufacturing to fabricate cutting tools. Hanzl et al. [25] designed a tool body to be 3D printed. The topological optimization involved reducing the tool weight to improve the dynamics of the cutting process and vibration absorption, and as a result, enhance the life of cutting edges. Lakner et al. [21] focus on supplying the cutting fluid through channels inside the tool. The geometry of the tool to be 3D printed was modified and then compared with that of a conventional tool. When this new type of tool was used for cutting AISI 4140+QT, the wear was lower and the tool vibration damping changed. The 3D printing of monolithic cutting tools was studied, for example, by Singh et al. [26] and Sandhu et al. [27]. The articles deal with the surface properties of expanded polystyrene (EPS) milled with a 3D printed monolithic tool made out of acrylonitrile butadiene styrene (ABS). From the literature on the subject, it is clear that fabricating cutting tools using additive manufacturing is becoming increasingly popular. The key parameters used to describe the cutting properties of such tools are surface roughness, runout, hardness and cutting edge radius.

This study was undertaken because no literature on the 3D printing of cutting tools or their dimensional and geometric accuracy was available.

From a review of the literature, it is evident that both laser cladding [28] and laser sintering may involve problems. Examples include microstructural defects, which may affect the mechanical properties of prints considerably. The results presented here will hopefully contribute to the development of a new enhanced 3D printing method for fabricating cutting tools, even on a mass scale.

The novelty of this study lies in applying powder-based 3D printing technology to create monolithic cutting tools. The article aims to assess whether 3D printing can be used for this purpose. The investigations were conducted for an end mill created using a Concept Laser M2 Cusing DMLM system. The material was steel 1.2709. As the condition of the cutting edge and its sharpness are some of the key factors in cutting operations [29,30], the analysis of the additively manufactured tool focused on determining its dimensional and geometric accuracy, the cutting edge radius and the surface quality.

## 2. Materials and Methods

### 2.1. Printing Method

The cutting tool under study was a milling cutter printed at the Radom Center for Innovation and Technology sp. z o.o. in Radom, Poland using a Concept Laser M2 Cusing Laser Powder-Bed system (Concept Laser GmbH, Lichtenfels, Germany).

**Figure 1:**
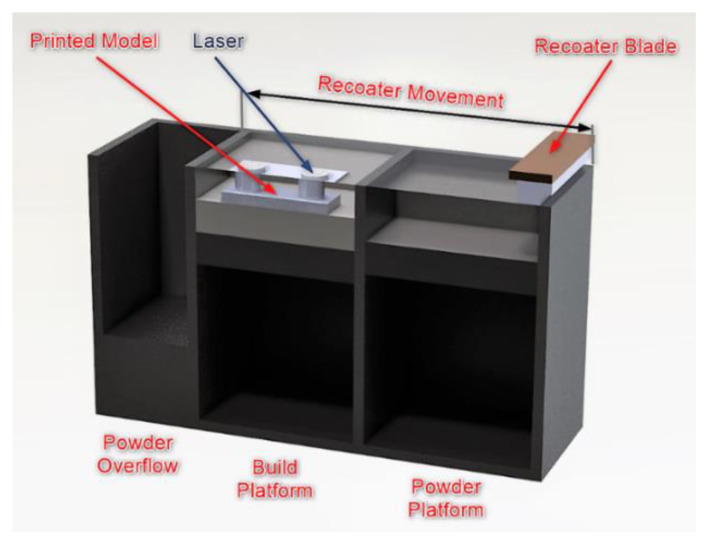
Schematic representation of the Concept Laser M2 Laser Cusing^®^ system.

A schematic diagram of the Concept Laser M2 Cusing system is shown in Figure 1. The build envelope is 250 × 250 × 280 mm (*x*, *y*, *z*). The system uses a continuous wavelength fiber laser source with a maximum variable output power of 200 W. The maximum scanning speed is 7 m/s; for variable focus move, it is 4.5 m/s. In the experiment, the focus diameter was 50 µm; however, the focus diameter may range between 50 and 500 µm.

The cutting tool was fabricated using argon as a shielding gas to keep oxygen level below 0.1% by volume. The layer thickness on the *z*-axis was 20 µm.

Figure 1 shows a diagram of a Concept Laser M2 Laser Cusing^®^. Its description is provided, for instance, in [31,32]. The principle of operation of this machine is similar to that of other 3D printers using powder materials. In this case, each layer of powder is first spread by the recoater and then melted selectively by the laser. The laser beam is directed by the computer-controlled mirrors. The resultant ultra-thin liquid pools solidify rapidly as they cool.

The Concept Laser M2 system uses a standard island-type scanning strategy. The area to be raster scanned is divided into small squares, the so-called ‘islands’. Each island is scanned by the laser spot in one direction, perpendicular to those in the neighboring islands. The selective melting of the material in the particular islands is done at random to prevent residual stress [31].

**Figure 2:**
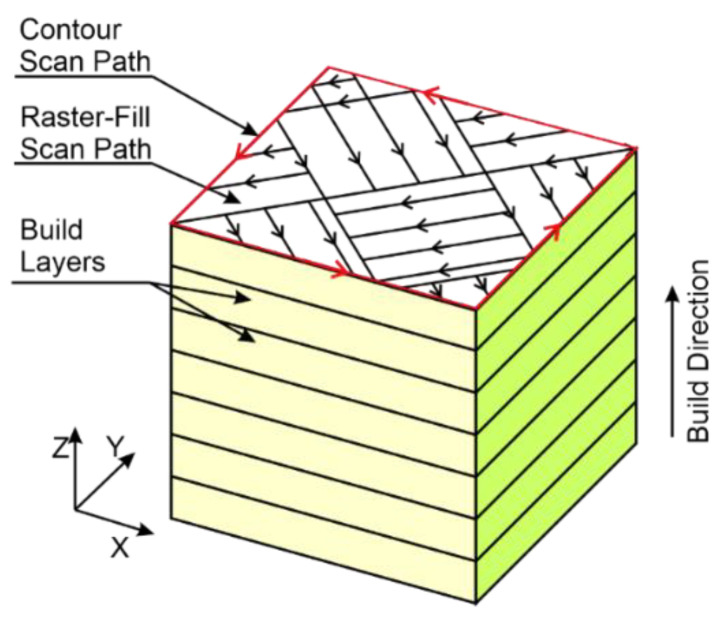
Schematic representation of the laser scanning strategy.

The scanning vectors are simple, alternating vectors. The distance between any two parallel vectors is defined as the scan spacing. The speed of the laser spot moving across the surface is referred to as the scanning speed. Neighboring islands have vectors directed perpendicular to one another. The ‘island’-type scanning strategy is represented schematically in Figure 2. All the islands are squares 5 mm by 5 mm in size. Once the selective melting of the islands is completed, the laser scans the area around the outer contour to finish the surface of a layer built. Each time a new layer is laid, the pattern of islands is shifted by 1 mm along the *x* and *y* axes to ensure that the island borders are not one over another to form ‘flaws’.

Table 1 shows the chemical composition of steel 1.2709.

The tool analyzed here was made of steel 1.2709, characterized by high yield strength, high tensile strength, and high impact resistance. Since the material is malleable and strong in a wide range of temperatures, it can be used for elements of a rocket engine.

### 2.2. Tool Fabrication

**Figure 3:**
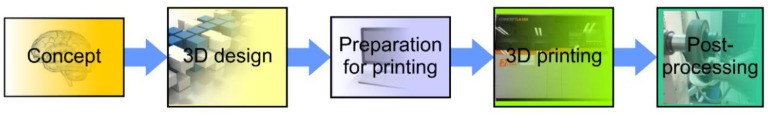
Stages of the tool development.

The cutting tool under analysis was fabricated using the DMLM additive manufacturing technology. The tool development process, illustrated in Figure 3, started with an idea, which was transferred into a 3D model using Siemens NX software (Version NX 9, Siemens PLM Software, Plano, TX, USA). This was followed by CAD validation to check the model geometry, surface area, etc. The next step was triangulation to guarantee geometric and angular accuracy of the object. Then, it was necessary to select the printing parameters such as build orientation, layer thickness, laser power, laser scanning speed, laser spot diameter, etc.

After the parameters were set, the printing began. The ready object was cleaned and finished by grinding. The finishing operations may also include coating.

The study entailed printing one monolithic tool standing vertically on the print bed. All the numerical data reported in this article are the arithmetical means of three measurements.

**Figure 4:**
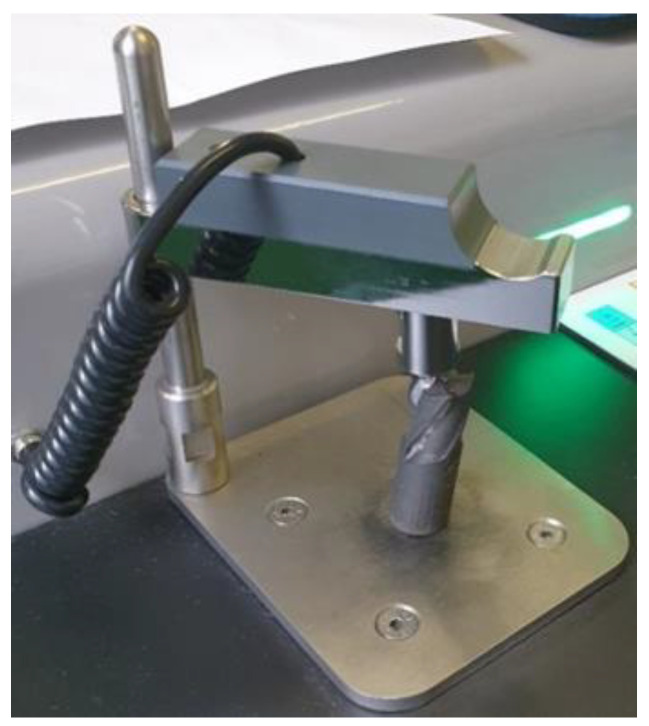
Chemical composition analysis of the printed tool.

The first analysis of the as-fabricated tool aimed to determine the chemical composition of the tool material. Figure 4 illustrates how the analysis was carried out.

The measurement was performed using an S3 MiniLab 300 spark spectrometer (G.N.R. S.r.l., Agrate Conturbia, Italy) which is a compact optical emission spectrometer for aluminum alloys, copper alloys and ferrous alloys.

### 2.3. Geometry and Dimensions

**Figure 5:**
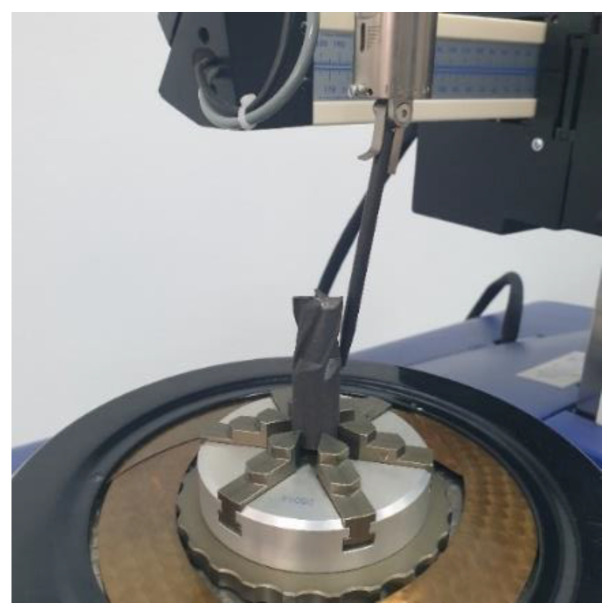
Measurement of the roundness and cylindricity profiles of the shank.

The shank is a very important part of the cutting tool, so if there are fabrication errors there, the radial runout of the tool may cause changes both in the feed and the forces acting on the operating tool. The shank of the printed end mill was ground to eliminate such problems. Prior to that, the shank roundness and cylindricity profiles were measured using a Talyrond 365 (Taylor-Hobson Ltd., Leicester, UK) system featuring a gauge with a range of up to 0.08 mm and a resolution of 1.3 nm and specialist software (Ultra roughness). The system offers high accuracy measurement. The surface parameters were determined on the basis of profiles measured using a Gaussian filter and by removing the waviness component using a 1-50 upr filter. The separation of two concentric circles that enclose the circular section is defined as Roundness (RONt). Figure 5 shows the 3D printed end mill mounted in the measuring system.

Measurements of the surface texture of the rake face were performed with a Taylor–Hobson Talysurf CCI Lite (Taylor Hobson Ltd., Leicester, UK) optical profiler fitted with a Mirau interferometer. The vertical resolution of the optical profiler was 0.01 nm, while the horizontal resolution was 0.33 μm. The surfaces was measured using a 20× zoom lens. The surface measured (0.8 mm × 0.8 mm) consisted of 1024 × 1024 measuring points. To determine the surface roughness parameters, 1024 profiles were filtered using a Gaussian filter (0.8 mm cut-off) and TalyMap Gold 6.0 software (Taylor Hobson Ltd., Leicester, UK). The measurements of the cutting edge radius were carried out using a 3D Keyence VR-5000 (Keyence Corporation, Osaka, Japan) optical profiler. The Keyence optical profiler is equipped with a VR-5200 40x magnification gauge. The area under analysis (7.6 mm × 5.7 mm) was measured with an accuracy of 0.5 μm. The device is equipped with a 4-megapixel monochrome Complementary Metal Oxide Semiconductor (CMOS) camera (1 inch and 4 million pixels).

The study also involved microscopic examination of the end mill structure using a JSM 7100F (Jeol Ltd., Tokyo, Japan) scanning electron microscope operated at an acceleration voltage of 15.0 kV, a probe current of 5 nA and a resolution of up to 3.0 nm.

## 3. Results and Discussion

### 3.1. Chemical Composition

The first tests aimed at determining the chemical composition of the tool material. Table 2 provides information about the chemical composition of the tool material, analyzed using the optical emission spectrometer. The weight percentages of the elements present in the print were compared with those of the powder material, i.e., maraging steel 1.2709 (available at the producer’s website). Generally, the amounts of the elements making up the print material fell within standard ranges; the only differences were observed for sulfur and silicon.

### 3.2. Cylindricity and Surface Texture

**Figure 6:**
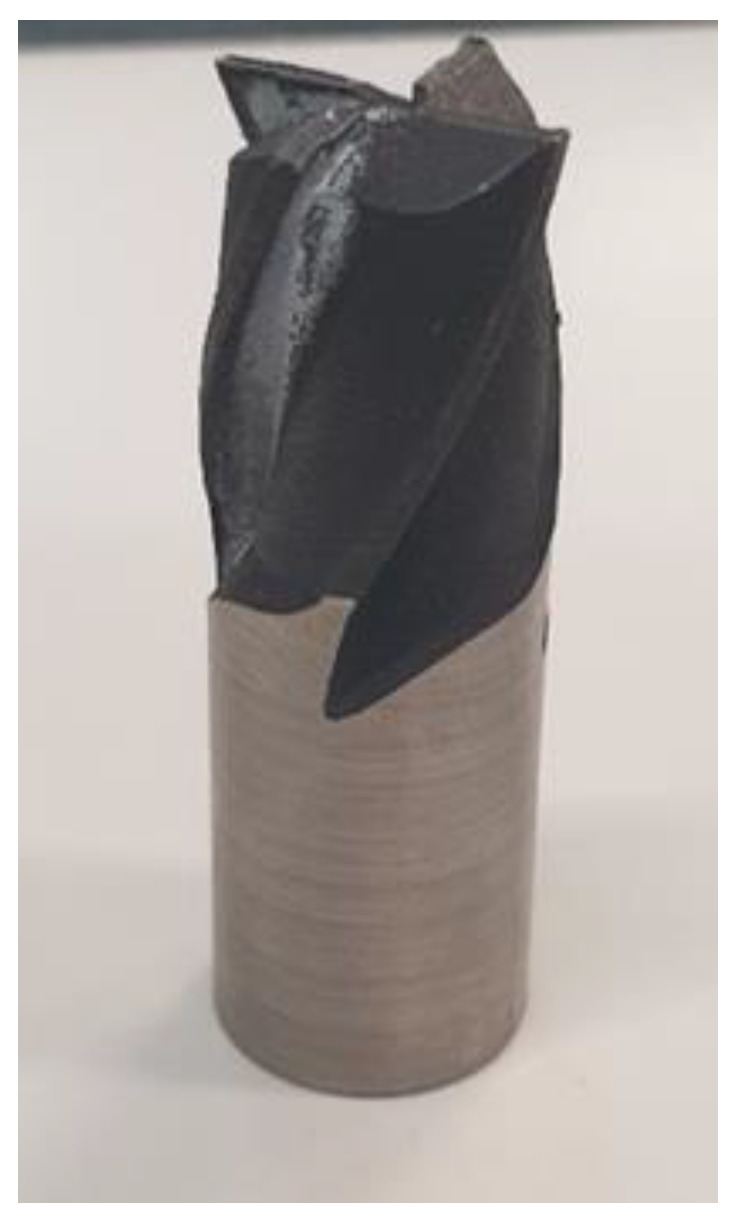
General view of the 3D printed tool.

Figure 6 shows a general view of the additively manufactured milling cutter. The tool shank required grinding to improve its mounting in measuring instruments, and this was done using a SAACKE UW I C (Gerb. Saacke GmbH & Co. KG, Pforzheim, Germany) grinding machine.

Looking at the printed tool with the naked eye, one can see that none of the cutting edges is sharp. This feature is a result of both the fabrication process and the particle size of the powder selected for the experiment.

**Figure 7:**
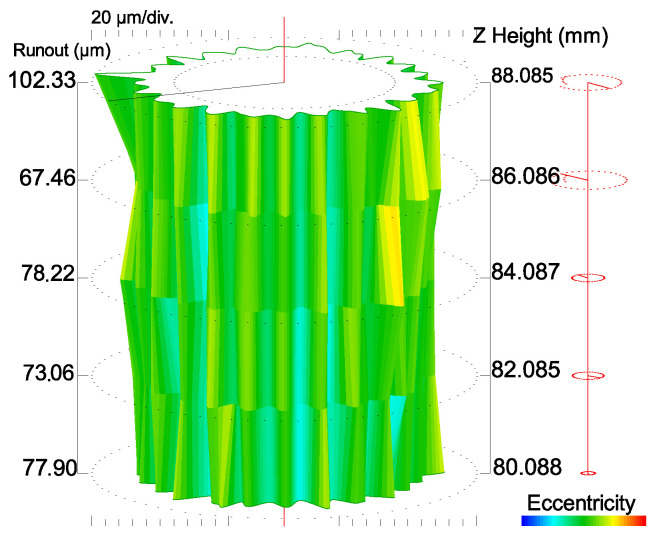
Determining the cylindricity of the tool shank.

Roundness measurements in five cross-sections with a Talyrond 365 system provided information about the shank cylindricity. The maximum and minimum local cylindricity errors were determined by comparing the cylindrical part of the printed tool to the reference cylinder. The peak maximum material departure from the reference cylinder (the CYLp parameter) was 76.37 μm, while the valley maximum departure from the reference cylinder (the CYLv parameter) was 43.17 μm. The cylinder taper (CYLtt parameter) was also measured, and it was 154.3°. From the point of view of practicality, the key parameters to describe the tool shank are the total runout and the taper angle. A high value of the taper angle may result in the need to point-mount the shank in the tool holder. The total runout of the cutting tool was 119.5 μm, whereas the taper angle was 0.19°. Since these values were not satisfactory, i.e., too high, the shank had to be ground before the tool could be used for cutting. The cylindricity measurement report is shown in Figure 7.

As shown in the article by Hanzl et al. [33], the cylindricity of the print is dependent on the angle at which the print is located on the bed. In the vertical orientation (i.e., the same as that analyzed in this article), the maraging steel 1.2709 specimens printed using direct metal laser sintering (DMLS) had a cylindricity of 72 μm. The result is similar to the value reported for the DMLM printed tool, i.e., 73.37 μm.

**Figure 8:**
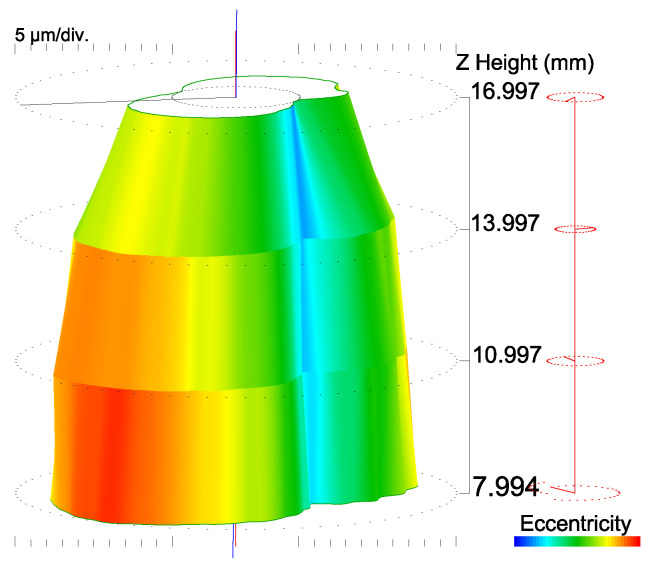
Cylindricity of the shank after grinding.

The grinding was performed using a Saacke UW I C grinding machine with the aim of reducing the registered errors. Figure 8 shows the cylindricity of the shank after grinding.

After grinding, the cylindricity parameters improved significantly. CYLp was reduced from 76.37 μm to 23.74 μm, and CYLv decreased from 43.17 μm to 25.95 μm. The total runout after grinding reached 49.7 μm.

**Figure 9:**
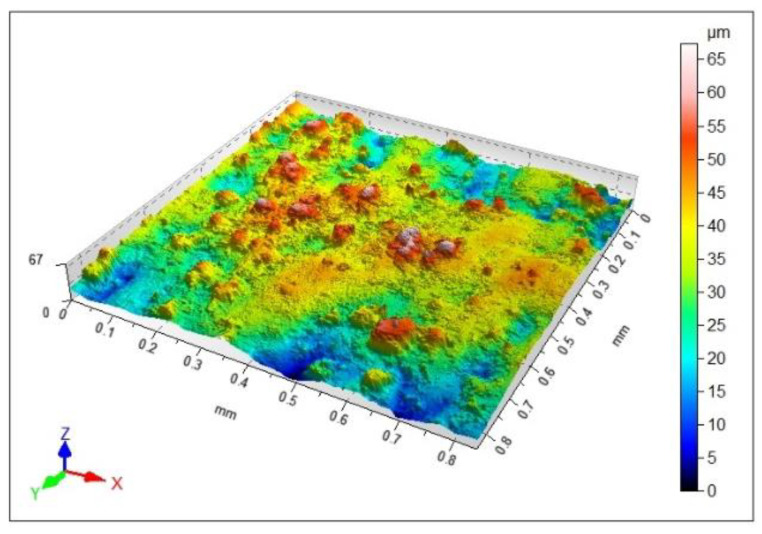
Surface texture measured on the rake face.

The quality of the printed tool surface was checked using an optical profilometer. Figure 9 shows an isometric 3D view of the surface. As can be seen from the image, there are areas of higher roughness (agglomerates), characteristic of selective laser sintering and direct laser deposition melting. The maximum height, represented by the Sz parameter, was reported to be 67 μm. The Sa parameter, i.e., the arithmetical mean height, was 7.74 μm, while the Sq parameter, i.e., the root mean square height, was 9.7 μm. Similar higher roughness areas (agglomerates) are described in [34]. The SLS-printed cuboid specimens made of maraging steel 1.2709 were found to have such higher roughness areas whatever their orientation on the print bed. The Sa parameter measured for a vertically SLS-printed specimen ranged from 7.01 to 8.41 μm. By comparison, Sa was 7.74 μm for the milling cutter analyzed here.

The measurements showed that the values of the height parameters were not satisfactory. The surface roughness had to be reduced before the tool was used, and that was achieved by grinding.

**Figure 10:**
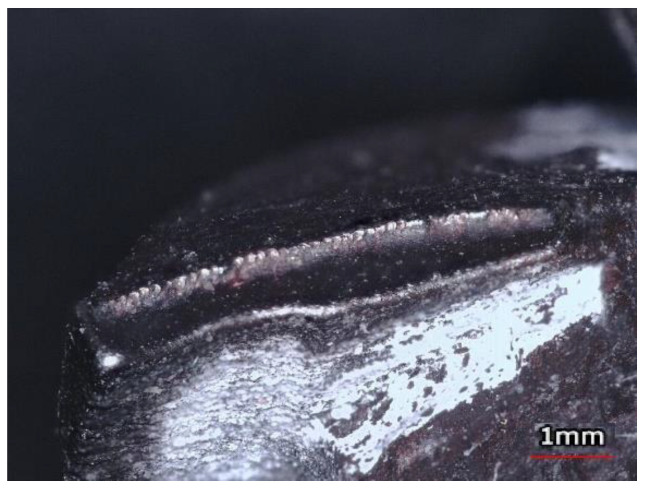
Rounded cutting edge of the as-fabricated tool.

Apart from the surface quality of the rake face, the other important parameters to be studied for a 3D printed milling cutter are the surface texture of the flank face and the cutting edge radius r_n_. Figure 10 illustrates the ‘rounded’ cutting edges of the as-fabricated end mill. Since there were no clear sharp edges, the cutting edge radius was measured.

**Figure 11:**
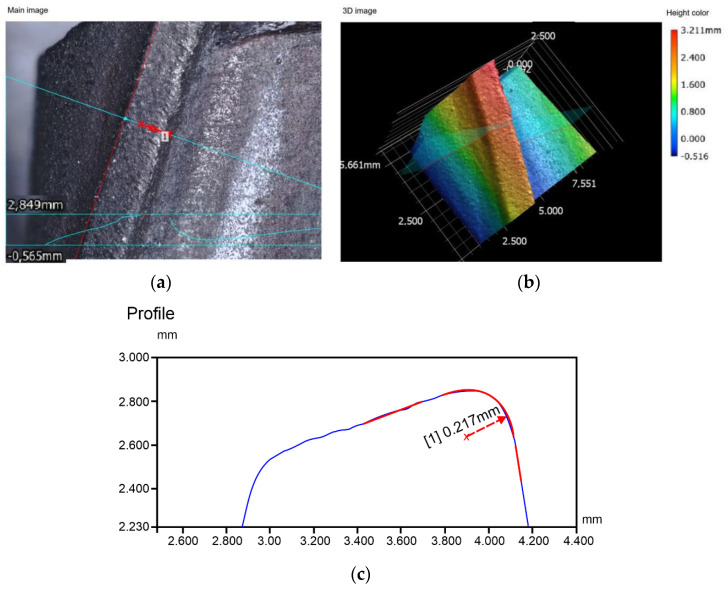
Measurement of the cutting edge radius, (**a**) scan image of the area measured, (**b**) digital image of the area measured, (**c**) edge profile.

Figure 11 shows the measurement report of the cutting edge radius. In Figure 11a, the line of cut is marked in red. Figure 11c depicts the cutting edge profile with a cutting edge radius of 217 μm. After grinding, the value dropped to 25 μm. For monolithic end mills, the cutting edge radius generally ranges from 30 μm to 60 μm.

The cutting edge radius is an important parameter in milling. Thus, before a 3D printed tool is used for cutting, it needs to be ground to produce sharp cutting edges. In the CAD model, the cutting edge radius was assumed to be zero. The literature on the subject does not discuss measurements of edge radius for additively manufactured tools.

**Figure 12:**
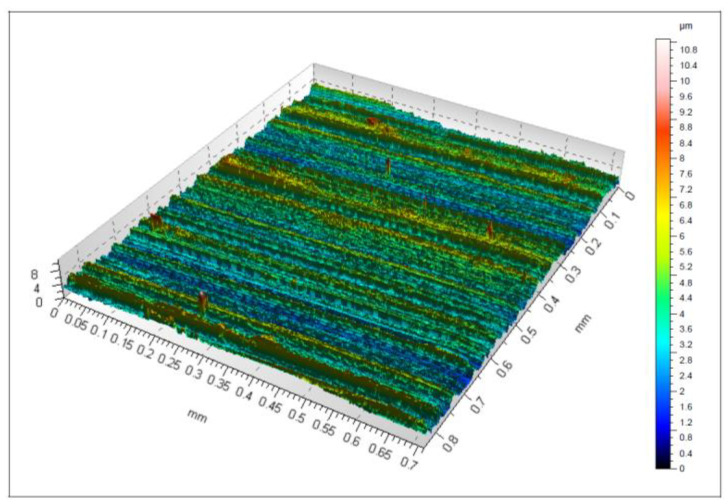
Surface texture of the rake face after grinding.

Additionally, the 3D Keyence VR-5000 optical profiler (Keyence Corporation, Osaka, Japan) was used to measure the Ra and Rz roughness parameters of the cutting edge, which were 4.6 μm and 23.5 μm, respectively. Surface roughness measurements were also carried out after grinding. The surface texture of the rake face after grinding is illustrated in Figure 12.

The surface quality of the printed tool after finishing is depicted in Figure 12. The agglomerates visible in Figure 9 were removed by grinding. Now, the surface contains marks of the finishing operation, i.e., parallel passes of the tool. The maximum height of the surface, Sz, was 11 μm. The Sa parameter representing the arithmetical mean height was 0.86 μm. The root mean square height, Sq, was 1.1 μm. In a study by Grobelny et al. [35], where the maraging steel 1.2709 specimens were fabricated using an SLS printer (Concept Laser M1) and finished by turning, the lowest value of the Sa parameter was 1.68 μm. This means that the value of Sa presented in this article is lower.

### 3.3. Microscopic Analysis

**Figure 13:**
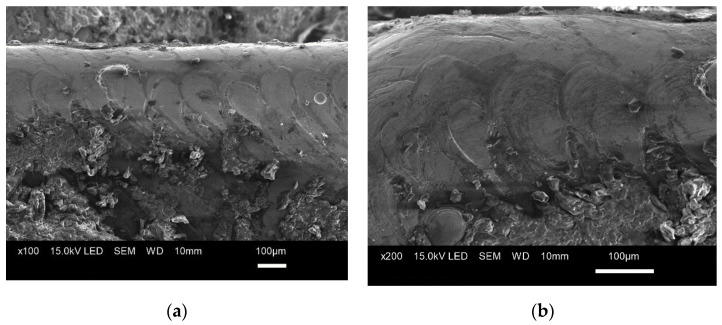
Tool structure magnified (**a**) 100×, (**b**) 200×.

The images in Figure 13 show the structure of the 3D printed tool with characteristic marks left by the laser beam. As can be seen, the structure is uniform; there are no flaws such as discontinuities, porosity, or cracks at the layer boundaries.

The structure of the tool is similar to that of thin-walled ceramic elements. In [36], Hu et al. focus on the enhancement of extrusion-based three-dimensional (3D) printing methods. The major aim of their paper is to present a new shaping retention method based on mathematical synthesis modeling for extrusion-based 3D-printing of ceramic pastes.

**Figure 14:**
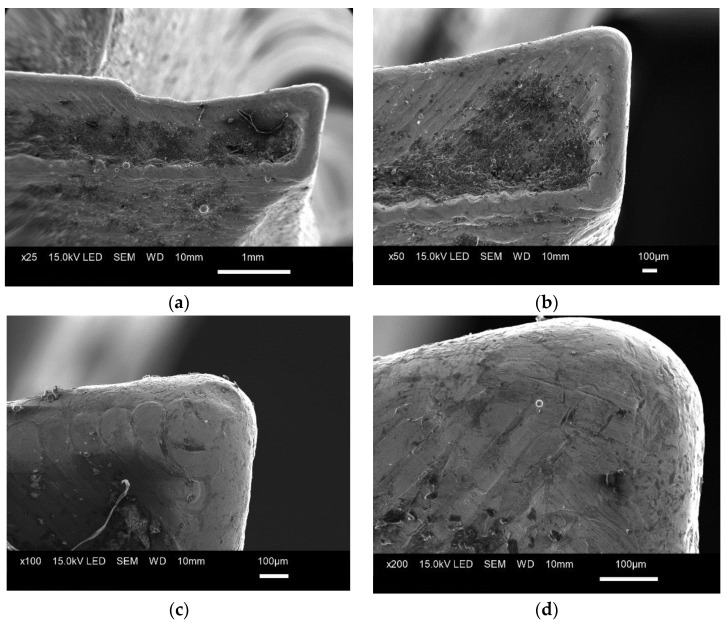
Microscopic images of the cutting edge magnified (**a**) 25×, (**b**) 50×, (**c**) 100×, (**d**) 200×.

The analysis of the rake and flank faces (Figure 13a,b) revealed the contour to be the distinctive feature of the structure. The cutting edges were rounded, not sharp, making the tool unsuitable for cutting (Figure 14).

The microscopic analysis confirmed that grinding was necessary to sharpen the cutting edges of the additively manufactured tool before it was used for cutting purposes.

**Figure 15:**
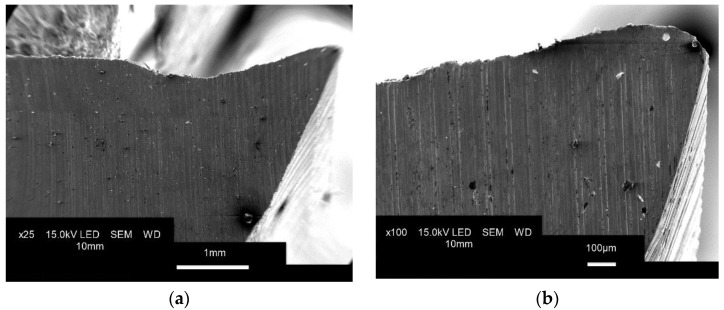

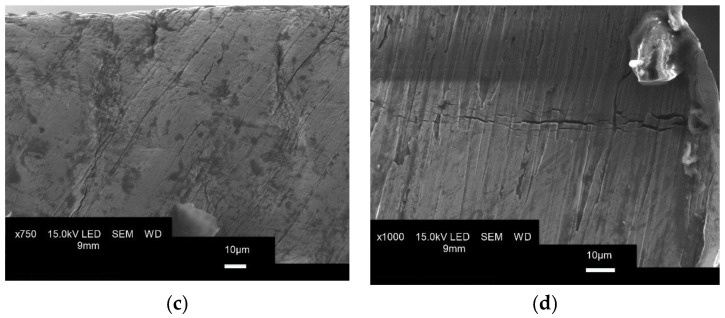
Microscopic images of the rake face magnified (**a**) 25×, (**b**) 100×, (**c**) 750×, (**d**) 1000×.

Figure 15 shows microscopic scans of the tool structure after grinding.

There are visible marks left by the grinding tool and characteristic voids after larger fragments of the surface material were removed during the process. At magnifications of 750× and 1000×, we can see material cracking, which may contribute to faster wear of the tool. Similar defects at the surface of maraging steel 1.2709 were observed by Živčák et al., for specimens printed using DMLS [37].

The lifespan of the cutting tool can be increased, firstly, by optimizing the additive manufacturing process, secondly, by heat treating and finishing the tool and, finally, by coating it. Another approach to ensure high quality of the tool is to optimize the design using CAD software. The importance of CAD modelling is described, for instance, in [38], but the tool was manufactured conventionally using a DMG MORI DMU 50 5-axis milling machine. Additive manufacturing offers greater flexibility with regard to the tool geometry, its mass reduction, or the location of channels through which cutting fluid is supplied.

## 4. Conclusions

The article has analyzed the potential use of additive manufacturing to fabricate milling cutters. The study involved printing an end mill using a 3D Concept Laser M2 system. The following are the main conclusions drawn from the experiments, which can be treated as initial suggestions for the design and fabrication of 3D printed milling cutters:

1. The difference between the 3D geometric model and the model created with a 3D Concept Laser M2 system ranges from 0.25 mm to 0.33 mm. It is thus recommended that machining allowance of a minimum of 0.5 mm should be planned at the geometry design stage.
2. The key geometric parameters to be taken into account during the design are: the shank cylindricity, the total runout of the shank, and the cutting edge radius.
3. The standard cutting edge radius should range between 30 μm and 60 μm. In the study, the cutting edge radius after printing was 217 μm. The grinding process reduced the cutting edge radius to 25 μm.
4. The measurements of the dimensional and geometric accuracy of the tool revealed that the total runout of the shank was 119.5 μm. After finishing, the total runout reached 49.7 μm.
5. After printing, the maximum height of the surface, Sz, was 67 μm. The Sa parameter, representing the arithmetical mean height, was 7.74 μm. After grinding, the two parameters were 11 μm and 0.86 μm, respectively.
6. The chemical composition of the milling cutter did not differ much from the composition of the powder material, with the exception of two elements, i.e., sulfur and silicon, whose percentages in the former case were slightly higher.

## Figures and Tables

**Table 1:** Chemical composition of maraging steel 1.2709 (powder).

Chemical Composition in wt. %														
Workpiece Material	Aluminum, Al	Carbon, C	Cobalt, Co	Chromium, Cr	Manganese, Mn	Molybdenum, Mo	Nitrogen, N	Nickel, Ni	Oxygen, O	Phosphorus, P	Sulfur, S	Silicon, Si	Titanium, Ti	Iron, Fe
Max	0.1	0.03	10	0.25	0.15	5.2	0.1	19	0.1	0.01	0.01	0.1	1.2	≈65
Min	-	-	8.5	-	-	4.5	-	17	-	-	-	-	0.8	

**Table 2:** Chemical composition of the end mill.

Chemical Composition in wt.%												
End mill Material	Aluminum, Al	Carbon, C	Cobalt, Co	Chromium, Cr	Manganese, Mn	Molybdenum, Mo	Nickel, Ni	Phosphorus, P	Sulfur, S	Silicon, Si	Titanium, Ti	Iron, Fe
	0.045	0.01	8.678	0.001	0.134	4.889	18.581	0.004	0.024	0.155	1.015	≈66.464

## Data Availability

Not available.
